# Novel repellents for the blood-sucking insects *Rhodnius prolixus* and *Triatoma infestans*, vectors of Chagas disease

**DOI:** 10.1186/s13071-020-04013-5

**Published:** 2020-03-18

**Authors:** Melanie Ramírez, Mario I. Ortiz, Pablo Guerenstein, Jorge Molina

**Affiliations:** 1 Centro de Investigaciones en Microbiología y Parasitología Tropical (CIMPAT), Departamento de Ciencias Biológicas, Universidad de los Andes, Bogotá, Colombia; 2 Laboratorio de Estudio de la Biología de Insectos, Centro de Investigación Científica y de Transferencia Tecnológica a la Producción (CONICET-Prov. Entre Rios-Uader), Diamante, Argentina; 3 Facultad de Ingenieria, Universidad Nacional de Entre Ríos, Concepción del Uruguay, Entre Rios Argentina

**Keywords:** Semiochemicals, *Citrobacter*, Skin microbiota, Volatile organic compounds, DEET

## Abstract

**Background:**

Studying the behavioral response of blood-sucking disease-vector insects to potentially repellent volatile compounds could shed light on the development of new control strategies. Volatiles released by human facial skin microbiota play different roles in the host-seeking behavior of triatomines. We assessed the repellency effect of such compounds of bacterial origin on *Triatoma infestans* and *Rhodnius prolixus*, two important vectors of Chagas disease in Latin America.

**Methods:**

Using an exposure device, insects were presented to human odor alone (control) and in the presence of three individual test compounds (2-mercaptoethanol, dimethyl sulfide and 2-phenylethanol, the latter only tested in *R. prolixus*) and the gold-standard repellent NN-diethyl-3-methylbenzamide (DEET). We quantified the time the insects spent in the proximity of the host and determined if any of the compounds evaluated affected the behavior of the insects.

**Results:**

We found volatiles that significantly reduced the time spent in the proximity of the host. These were 2-phenylethanol and 2-mercaptoethanol for *R. prolixus*, and dimethyl sulfide and 2-mercaptoethanol for *T. infestans*. Such an effect was also observed in both species when DEET was presented, although only at the higher doses tested.

**Conclusions:**

The new repellents modulated the behavior of two Chagas disease vectors belonging to two different triatomine tribes, and this was achieved using a dose up to three orders of magnitude lower than that needed to evoke the same effect with DEET. Future efforts in understanding the mechanism of action of repellent compounds such as 2-mercaptoethanol, as well as an assessment of their temporal and spatial repellent properties, could lead to the development of novel control strategies for these insect vectors, refractory to DEET.
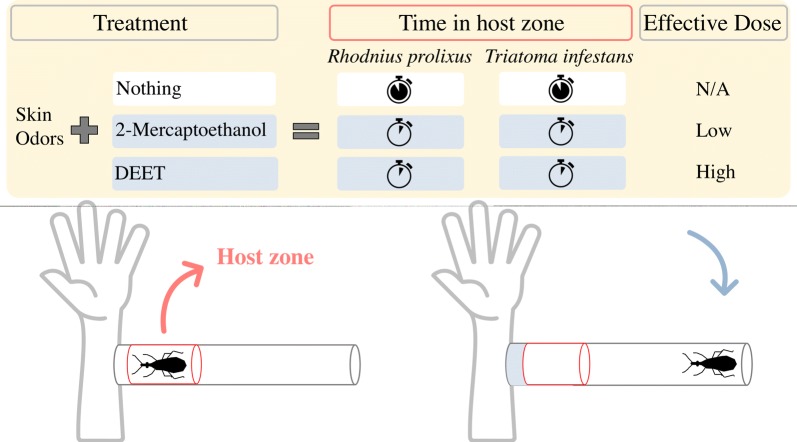

## Background

Most vectors of human infectious diseases are bloodsucking insects, and therefore, many of those diseases could be managed by the use of insect-vector control strategies [1]. For example, it is strongly advised that people living in or visiting regions populated by insects that feed on blood, such as mosquitoes, protect themselves using insect repellents [2]. Independently of its mechanism of action, the final effect of a repellent is to cause an insect to make oriented movements away from its source. The expected result is to disrupt the host-seeking behavior of the threatening insect [3–5].

Triatomine bugs (Hemiptera: Reduviidae: Triatominae) feed on the blood of vertebrates and are vectors of the protozoan parasite *Trypanosoma cruzi*, the etiological agent of Chagas disease, also known as American trypanosomiasis [6]. The vast majority of the extant 149 species of triatomines are found in Latin American countries, where 68 triatomine species have been found infected with *T. cruzi*, and more than 150 species of domestic and wild mammals have been found to carry the parasite [7–12]. However, few triatomine species are recognized as competent vectors, and only approximately five species are considered very important vectors for humans: *Rhodnius prolixus* Stål, 1859 (inhabiting mainly Colombia and Venezuela), *Triatoma infestans* (Klug, 1834) (inhabiting mainly Peru, Bolivia, Paraguay and Argentina), *T. dimidiata* (Latreille, 1811) (inhabiting Mexico and Central America), *T. brasiliensis* Neiva, 1911 and *Panstrongylus megistus* (Burmeister, 1835) (both found mainly in Brazil) [8, 13]. Although there are many routes of *T. cruzi* transmission (i.e. oral, blood transfusion, mother to child), the vectorial infection can occur if, after taking a large blood meal, the insect defecates on the host skin and the feces carrying infective forms of *T. cruzi* enter the blood stream through the wound or any mucous tissue [8]. Since its discovery by Carlos Chagas, controlling vectorial transmission has been the most suitable method to prevent Chagas disease, which affects approximately 7 million people worldwide [14].

Historically, most research on repellents has focused on mosquitoes over other blood-sucking arthropods such as triatomines [4, 15–21]. This tendency to focus on mosquito-repellent research is not surprising considering the higher mortality and morbidity due to mosquito-borne diseases compared to that of Chagas disease [22–24]. For almost six decades, NN-diethyl-3-methylbenzamide, known as DEET, has been the most common mosquito repellent used worldwide [25]. In fact, the effectiveness of DEET against all groups of biting arthropods, triatomines included, has granted it the title of the gold standard among repellents [4, 5]. However, compared with mosquitoes and other blood-sucking arthropods, triatomines have a lower sensitivity to this repellent [19, 26]. Studies with *R. prolixus* and *T. infestans* have revealed that whether the host is present or not, only high doses (i.e. > 90%) have a repellent effect, making DEET rather impractical for reducing human-triatomine contacts [15, 27–30]. In addition to these and other related findings in triatomines (i.e. DEET pre-exposure adaptation, DEET repellency in pyrethroid resistant colonies and the effect of nitric oxide on the sensory detection of DEET) [20, 31, 32], other studies have explored natural repellents such as essential oils, aiming at finding alternatives to DEET and other synthetic repellents [18, 21, 23, 33–36].

A decade of research has shown that volatile organic compounds (VOCs) from human skin and of microbial origin play a role in the behavioral responses of some blood-sucking insects [37]. For example, VOCs produced by skin bacteria are important cues for the malaria vector *Anopheles gambiae* to identify hosts as human and even to confer specificity to certain body regions on which mosquitos tend to bite more [37–42]. Moreover, previous studies carried out in our laboratory have demonstrated the role that VOCs released by human facial skin microbiota play in the host-seeking behavior of *R. prolixus* [43–45]. Thus, Tabares et al. [43] showed, in dual choice olfactometer experiments, that VOCs produced *in vitro* by some skin bacteria (at specific growth phases) have an attractive effect on *R. prolixus*. The authors also reported odor-source avoidance when some other bacteria VOCs were presented, such as those produced by *Citrobacter koseri* (*Enterobacteriaceae*). Thus, in this case, insects consistently chose the negative control (i.e. culture medium without bacteria) over the culture medium with bacteria VOCs. These two findings, the attractive and avoidance behavioral effects, contrast with those obtained using even other bacterial VOCs to which *R. prolixus* did not respond at all [43].

These studies show that the behavioral response of triatomines to the mix of VOCs produced by the skin microbiota is very complex [43]. Moreover, the role of individual bacterial volatiles from mixtures evoking avoidance is still unknown, and their potential use as repellents deserves further investigation. In this study, we asked whether individual VOCs released by cultures of *C. koseri*, which evokes avoidance, could affect the behavior of kissing bugs in the proximity of a human host causing, for example, a repellent effect. Furthermore, we investigated whether this potential effect could be equivalent to that evoked by the well-known repellent DEET. Thus, using an exposure device, we investigated in *R. prolixus* and *T. infestans* the repellent effect of three compounds which are structurally similar to compounds identified from cultures of *C. koseri* [43]; 2-mercaptoethanol, 2-phenylethanol and dimethyl sulfide. We compared the repellency effectiveness of these compounds at different doses with that obtained with DEET.

## Methods

### Insects

Adults of *R. prolixus* and third-instar nymphs of *T. infestans* from our laboratory colonies were used (the reason for using different life stages for the two species relates to insect availability). The *R. prolixus* colony originated from wild populations from San Juan de Arama, Meta Department (Northeast of Colombia), and has been maintained at the Centro de Investigaciones en Microbiología y Parasitología Tropical (CIMPAT), Universidad de los Andes (Bogotá, Colombia) since 1979, while the *T. infestans* colony originated from wild populations from Chaco Province (Northeast of Argentina; provided by the Centro de Referencia de Vectores, CeReVe-Argentina), has been maintained at the Centro de Investigacion Cientifica y de Transferencia Tecnologica a la Produccion (CICyTTP, Diamante, Argentina) since 2011, and has been receiving new wild insects from the same region almost every year for the last four years. Insects were fed on hens every two weeks and maintained under an artificial 12:12 h (light:dark) illumination regime at a controlled temperature and humidity (27 ± 2 °C, 75 ± 10% RH).

For experiments, insects were separated from the colony after molting and starved for at least 20 days for *R. prolixus* and 30 days for *T. infestans*. Experiments were video recorded using a DCR-SR 200 camera (Sony Corp., Tokio, Japan) or an A1633 iPhone camera (Apple Inc., Cupertino, USA) and performed during the early scotophase at 24.5 ± 0.5 °C in a dark (or red-light illuminated) room. Experiments with *R. prolixus* were performed at CIMPAT, Universidad de los Andes, and experiments with *T. infestans* were carried out at Laboratorio de Estudio de la Biología de Insectos (LEBI), CICyTTP. Insects were tested individually and used only once.

### Repellency tests

**Fig. 1:**
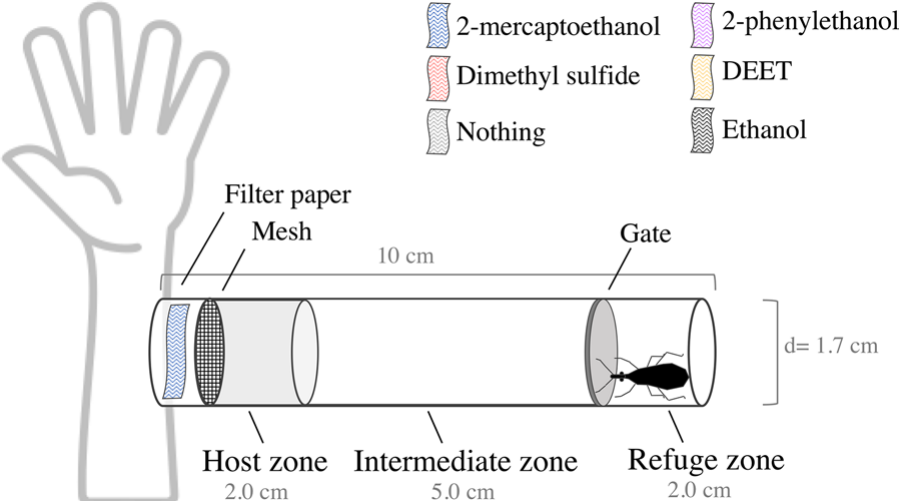
Exposure device with live host to test repellent effect of different compounds on *Rhodnius prolixus* and *Triatoma infestans*. Drawing not to scale. A polystyrene tube (10.0 × 1.7 cm, length × diameter, respectively) was divided in three zones: host, intermediate and refuge zone. At the end of the host zone a mesh allows transmission of stimuli released by the host (including VOCs alone or with repellent compounds), but avoid that insects could bite the host

To test the effect of structurally similar compounds produced *in vitro* by bacteria previously isolated from human facial skin [43], an exposure device modified from Zermoglio et al. [15] was used and is shown in Fig. 1. In brief, a polystyrene tube was divided into three zones: host, intermediate and refuge zones. An insect was placed in the refuge zone, and after a 5 min adaptation time, the experiment started with the opening of a gate, allowing the insect to freely move from the refuge to the other two zones. Insects attracted by the stimuli from the forearm of a volunteer walked to the host zone, while mesh prevented them from biting the volunteer. Experiments lasted 5 min. The exposure device allowed us to quantify the time the insect spent near the host in the presence or absence of the compounds tested.

To avoid different VOC profiles, we always tested the same forearm of only one volunteer. During the period of time that experiments were carried out, the volunteer was asked to avoid to use any soap when taking a shower, to refrain from drinking alcohol or eating any spicy food and using any perfumed cosmetics or any skin products. The volunteer does not smoke, was free from chronic illnesses and not using any medication on a regular basis.

Ten insects per treatment were used; these were randomly assigned to each treatment. Treatments for experiments with *R. prolixus* consisted of increasing concentrations (vol/vol) of 2-mercaptoethanol (0.0015625%, 0.003125%, 0.00625%, 0.0125%, 0.025%, 0.05% and 0.1%), dimethyl sulfide (0.00625%, 0.0125%, 0.025%, 0.05% and 0.1%), 2-phenylethanol (0.025%, 0.05%, 0.1% and 0.2%), and DEET (10%, 50% and 90%). Treatments for experiments with *T. infestans* consisted of increasing concentrations (vol/vol) of 2-mercaptoethanol (0.00625%, 0.025%, 0.1% and 1%), dimethyl sulfide (0.1% and 1%), and DEET (90%). The tested compounds were ≥ 99% pure (Merck, Darmstadt, Germany), while DEET was > 97% pure (Sigma-Aldrich, Darmstadt, Germany). Dimethyl sulfide and 2-mercaptoethanol solutions were diluted in distilled water, while 2-phenylethanol and DEET were diluted in ethanol. We performed frequent control tests before the beginning of treatment tests: host stimuli without any test compound (“host alone” a to d, see below) and host stimulus plus just ethanol (“host plus ethanol”, see below). The test odor stimulus consisted of a 10 μl solution (or just solvent for the controls) loaded onto a filter paper strip (1.0 × 3.0 cm). In the case of DEET, 10 μl or 50 μl solutions (where indicated) were used. The paper strip with the test solution or solvent control was carefully placed in the space between the host’s forearm and the mesh in the tube. Neither the host’s skin nor the insects were in direct contact with the compounds tested.

### Data analysis and statistics

We carried out nonparametric statistical tests to determine whether the compounds influenced the time that the insect spent in the host proximity. Prism software (GraphPad, v. 7.0a) was used to perform Kruskal–Wallis ANOVAs. Significant results (*P* < 0.05) were followed by Dunnett’s tests to compare the responses of each group with all other groups.

## Results

In this study, we assessed the repellency of VOCs released by the skin bacterium *C. koseri* against *R. prolixus* and *T. infestans*. For this, 240 starved adult *R. prolixus* and 90 starved *T. infestans* nymphs were assayed.

**Fig. 2:**
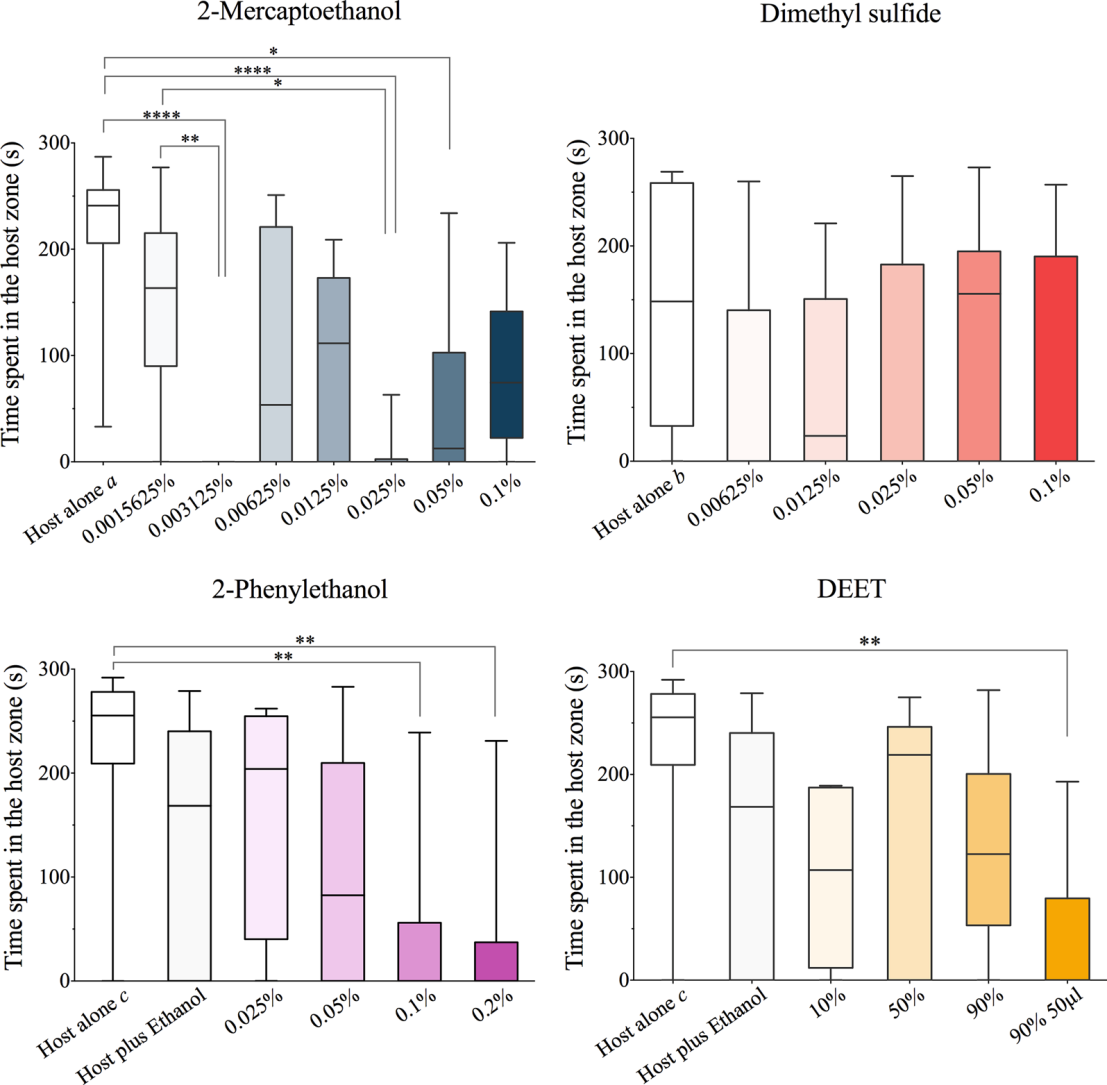
Box plots showing the effect of different doses of the test compounds on the time that *Rhodnius prolixus* spent in the proximity of a vertebrate host when the insects were exposed to 2-mercaptoethanol, dimethyl sulfide, 2-phenylethanol, and DEET (median, 25th and 75th percentiles are shown; whiskers denote minimum and maximum values). Asterisks denote significant differences among treatments according to Dunnett’s multiple comparison test (*P < 0.05; **P < 0.01, ****P < 0.0001). Host alone a, b and c are control repetitions consisting of exposure to the forearm of the host in the absence of any test compound

**Fig. 3:**
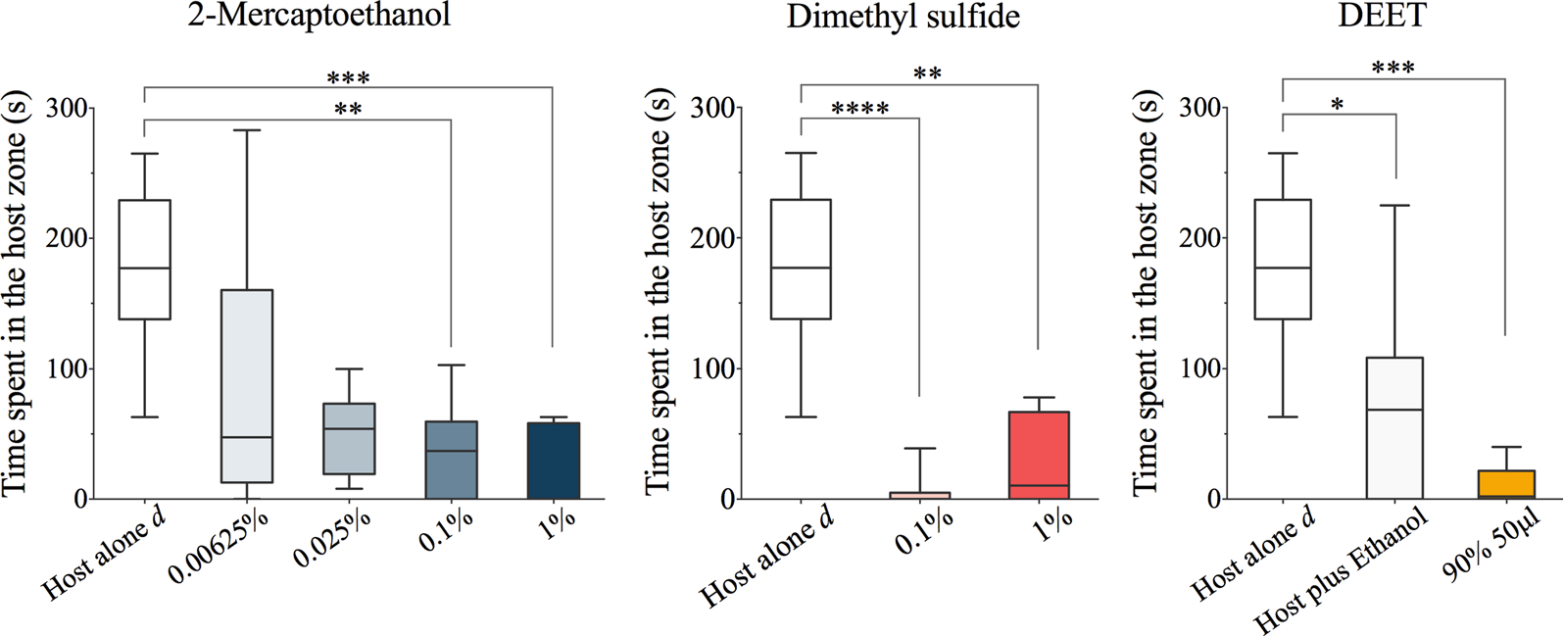
Box plots showing the effect of different doses of the test compounds on the time that *Triatoma infestans* spent in the proximity of a vertebrate host when the insects were exposed to 2-mercaptoethanol, dimethyl sulfide, and DEET (median, 25th and 75th percentiles are shown; whiskers denote minimum and maximum values). Asterisks denote significant differences among treatments according to Dunnett’s multiple comparison test (*P < 0.05; **P < 0.01, ***P < 0.001, ****P < 0.0001). Host alone d refers to a control consisting of exposure to the forearm of the host in the absence of any test compound

In the absence of test compounds, *R. prolixus* spent 81.6% (“host alone” a), 49.5% (“host alone” b), and 85.2% (“host alone” c) of the total experimental time within the host zone (Fig. 2, white boxes). In the case of *T. infestans*, insects spent 59% (“host alone” d) of the total time in the host zone (Fig. 3, white boxes). However, when certain doses of 2-mercaptoethanol, 2-phenylethanol or DEET were added, the time that adult *R. prolixus* spent in the host zone was significantly lower (Kruskal–Wallis H-test: *χ*^2^ = 38.29, *df* = 7, *P* < 0.0001; *χ*^2^ = 21.4, *df* = 5, *P* = 0.0007; *χ*^2^ = 17.48, *df* = 5, *P* = 0.0037, respectively) (Fig. 2). Likewise, certain doses of 2-mercaptoethanol and DEET considerably reduced the time that *T. infestans* nymphs stayed near the host (Kruskal–Wallis H-test: *χ*^2^ = 22.25, *df* = 4, *P* = 0.0002 and *χ*^2^ = 17.04, *df* = 2, *P* = 0.0002, respectively) (Fig. 3). It should be noted that Dunnett’s multiple comparison tests showed no differences between the times in the host zone for treatments in which the compounds were dissolved in ethanol and those for the control “host plus ethanol”. However, a significant difference was found when comparing the effect of the test compounds with that of host alone (Table 1).

**Table 1:** Summary of the multiple comparisons tests that resulted in statistically significant differences (*P* < 0.05), showing treatments that reduced the time the insects spent in the host zone with respect to the controls

Chagas disease vector	VOC tested	Kruskal–Wallis test	Comparison test	Dunnʼs multiple test *P*-value	Effective dose
*R. prolixus*	2-mercaptoethanol	< 0.0001	Host alone a *vs* 0.003125%	< 0.0001	Low
			Host alone a *vs* 0.025%	< 0.0001	
			Host alone a *vs* 0.05%	0.0148	
			0.003125% *vs* 0.0015625%	0.0081	
			0.025% *vs* 0.0015625%	0.0392	
	2-phenylethanol	0.0007	Host alone c *vs* 0.1%	0.0036	Low
			Host alone c *vs* 0.2%	0.0033	
	DEET	0.0037	Host alone c *vs* DEET 90% 50 µl	0.0010	High
*T. infestans*	2-mercaptoethanol	0.0002	Host alone d *vs* 0.1%	0.0019	Low
			Host alone d *vs* 1%	0.0001	
	Dimethyl sulfide	< 0.0001	Host alone d *vs* 0.1%	< 0.0001	Low
			Host alone d *vs* 1%	0.0058	
	DEET	0.0002	Host alone d *vs* host plus ethanol	0.0346	High
			Host alone d *vs* DEET 90% 50 µl	0.0001	

The time spent by *R. prolixus* near the host did not differ statistically from the control when dimethyl sulfide was tested (Kruskal–Wallis H-test: *χ*^2^ = 8.282, *df* = 5, *P* = 0.1414). In contrast, dimethyl sulfide did reduce the time that the *T. infestans* nymphs spent near the forearm (Kruskal–Wallis H-test: *χ*^2^ = 21.05, *df* = 2, *P* < 0.0001). A summary of the statistically significant results of the multiple comparisons tests is shown in Table 1.

## Discussion

Our results provide evidence that some VOCs released by the opportunistic skin bacterium *C. koseri* interfere with the host-seeking behavior of *R. prolixus* and *T. infestans*, two important vectors of Chagas disease. In control tests where just a host is presented, *R. prolixus* adults and *T. infestans* nymphs move their antennae in a triangulation fashion [46, 47], and in just a few seconds, usually walk towards the host, extend their proboscis and try to bite the forearm. However, when the compounds tested are added to the stimuli of the host, the behavior of the bugs changes; the time spent near the human host is considerably reduced (Figs. 2, 3). An additional movie file (Additional file 1: Video S1) shows that both species rapidly walk away from the stimulus source after approaching it, which suggests a clear repellent effect of these compounds (even when attractive stimuli such as heat and host VOCs are present). The methodology used in this work (based on that by Zermoglio et al. [15]) provides a fast and direct way to test the effect of candidate repellent compounds near a vertebrate host.

As it has been shown for mosquitos, *R. prolixus* is attracted by some VOCs released by human face skin microbiota [43–45]. These results provide further support for the hypothesis that the host-seeking behavior of triatomines is actually a tripartite relationship (host, vector and microbiota) and could be the result of the close vertebrate-vector coevolutive history. The observed repellency to VOCs produced by *C. koseri* may be understood if the natural occurrence of the bacterium is considered: *C. koseri* is a gram-negative bacillus of the family *Enterobacteriaceae* commonly found in animal intestines, soils, water, sewage and contaminated food, and it is widely recognized for causing devastating meningitis in neonates and severe infections in immunosuppressed patients [48]. This bacterium is not part of the healthy human skin microbiota; human skin isolations where this bacillus is found are commonly from sick patients [48, 49] so that, these volatiles could signal an unhealthy individual to the bugs.

Interestingly, it is not new that the VOC signature of the genus *Citrobacter* influences the chemotactic orientation behavior of blood-seeking insects. Ponnusamy et al. [50] found that VOCs released by *C. freundii* were attractive to gravid females of *Aedes* (*Stegomyia*) *aegypti* and *Ae.* (*Stegomyia*) *albopictus*, two mosquito species which are important vectors of arboviruses [51]. It was also suggested that *Citrobacter* VOCs, in synergy with other compounds present in water, give mosquitos information about the quality of the oviposition sites [50]. In the bloodsucking stable fly *Stomoxys calcitrans*, Romero et al. [52] showed that VOCs released by *C. freundii* induce oviposition in soil. Therefore, VOCs released by *Citrobacter* sp. appear to be an interesting semiochemical source, mediating interactions with biotic (e.g. animal and human hosts) and abiotic (e.g. water and soil) factors, which is crucial for insects of medical importance [53–56].

The VOC mix released by *Citrobacter* sp. can be described as having a strong, fetid and putrid odor. Many species among the genus are cataloged within the malodor-generating bacteria group, in part because of their participation in decomposition processes [57–59]. The compounds methanethiol and dimethyl disulfide, identified as VOCs released by *C. koseri* [43], and the two VOCs used in our study, 2-mercaptoethanol and dimethyl sulfide, are sulfur-containing compounds. Sulfur compounds are neurotoxic and lethal to some insects and are proposed as a new control alternative to agricultural pests [60, 61]. However, during our tests we did not observe any symptoms of intoxication (i.e. insects with abnormal rest positions, paralysis in the legs, or death [19]) due to sulfur compounds perhaps because of the low doses tested or because the insect never got in direct contact with the compounds tested. Nevertheless, the effects of these sulfur compounds on development, hatching, oviposition or molting of insects, sensory adaptation, or the toxicity to vertebrate animals and humans should be studied for future applications.

Both sulfur compounds, together with 2-phenylethanol, are also known and used as VOC markers of human and animal wastes [62, 63]. They are also involved in the decomposition of mammal and bird tissues [64, 65], a scenario that is probably not attractive to triatomine insects if that change host volatile profiles of diseased hosts despite the presence of other cues such as temperature and CO_2_. It should be noted that in this work, the time spent in the host zone when presenting 2-phenylethanol was significantly lower than that of the “host alone” control but not different from the “host plus ethanol” solvent control. Additionally, there were no significant differences between the two controls. This suggests that the repellent effect of 2-phenylethanol is evident only when presented together with ethanol. It is interesting to note that 2-phenylethanol is also produced by the Brindley’s glands of *T. infestans*, which are involved in the production of alarm pheromones in adult insects [66–68]. However, this compound has not been reported as part of the alarm pheromone of *R. prolixus* [68, 69]. Likewise, in *An. gambiae*, this compound was reported as a spatial repellent candidate that inhibits attraction [70, 71]. The effect that this compound could have on the behavior of *T. infestans* needs to be further assessed.

In this study, DEET was used as a repellent control. Here, the repellency effect of DEET for *R. prolixus* may be the result of an additive effect or synergy between the solvent and DEET, as in the case of 2-phenylethanol. Such a repellency effect of DEET (plus ethanol) was only achieved at the highest dose tested (i.e. 90%, 50 μl). In contrast, 2-phenylethanol (for *R. prolixus*), dimethyl sulfide (for *T. infestans*) and 2-mercaptoethanol (for both species) showed a repellent effect at doses two to three orders of magnitude lower than the effective dose of DEET (i.e. 0.003125–0.1%). Efficiency at low doses is one of the key characteristics that is required for a good, new repellent [25]. The need to employ high concentrations of DEET to achieve repellency has limited its application in disrupting triatomine-human contacts, as several studies have already shown [15, 27–30]. Although its use is deemed safe, DEET has some disadvantages: it needs to be constantly reapplied; it has a short range of action due to its low volatility and can melt plastics and vinyl [4, 25]. Even more important, the people who truly need it usually cannot afford it [4]. Repellents for triatomines that are alternative to DEET have recently been proposed [27], including 4-methylcyclohexanol, a compound that somewhat resembles 2-phenylethanol. In this work we add up to the list of novel candidate repellents for triatomines.

The question of why triatomines are almost refractory to the gold standard DEET is still open. One hypothesis concerning the repellent effect of DEET is that it mimics a defensive compound of plants, methyl jasmonate, which might explain why this compound is still effective in insects with an evolutionary association with plants, such as mosquitoes [4, 72]. Although some triatomine species such as *R. prolixus* have a close relationship with palm tree niches [73], molecules as DEET may not be directly related to the triatomine evolutive history as early ancestors of the Triatominae subfamily were predators, unlike plant-feeder mosquito ancestors. In fact, triatomines are obligate hematophagous, and many species have nearly no contact with plants [30, 73–75]. Regarding the mechanism of action of synthetic volatiles such as DEET, it has recently been proposed that those compounds decrease the amount of host volatiles reaching the olfactory neurons, changing the chemical profile of hosts [76]. Despite the advances in research on repellency in mosquitoes, where DEET is considered the gold standard, finding efficient repellents for triatomines still represents a challenge and deserves further investigation.

## Conclusions

To the best of our knowledge, this is the first study in triatomines that assesses the repellent effect of individual volatiles of microbial origin from a human host. We showed that vectors of two different tribes (Rhodniini and Triatomini), with epidemiological importance in Chagas disease transmission, are repelled by very low doses of the sulfur compound 2-mercaptoethanol. Future studies should be directed to understand deeply its mechanism of action in triatomines and to assess its possible use as a repellent (although not applied directly onto the skin) maybe within a push-pull vectorial control strategy.

## Supplementary information

**Additional file 1: Video S1.** Video recording showing the repellency effect of the tested compounds on *R. prolixus* and *T. infestans*.

## Data Availability

The datasets used and/or analysed during the study are available from the corresponding author upon reasonable request.

## Abbreviations

VOC: Volatile organic compound
DEET: NN-diethyl-3-methylbenzamide
